# Analyzing the Association of Social Capital and Sleep Quality of the Elderly Community

**DOI:** 10.1002/hsr2.70766

**Published:** 2025-05-19

**Authors:** Maryam Mohammadi, Sahar Mohammadnabizadeh, Mehdi Varmaghani, Mitra Moodi, Farshad Shrifi, Matin Shirazinia, Hossein Fakhrzadeh, Moloud Payab, Masoumeh Khorashadizadeh, Shohreh Naderimagham

**Affiliations:** ^1^ Social Determinants of Health Research Center Mashhad University of Medical Sciences Mashhad Iran; ^2^ Department of Health Education and Health Promotion, School of Health Mashhad University of Medical Sciences Mashhad Iran; ^3^ Department of Management Sciences and Health Economics, School of Health Mashhad University of Medical Sciences Mashhad Iran; ^4^ Geriatric Health Research Center Birjand University of Medical Sciences Birjand Iran; ^5^ School of Health, Department of Health Promotion and Education, School of Health Birjand University of Medical Sciences Birjand Iran; ^6^ Elderly Health Research Center, Endocrinology and Metabolism Population Sciences Institute Tehran University of Medical Sciences Tehran Iran; ^7^ Department of Medicine Mashhad University of Medical Sciences Mashhad Iran; ^8^ School of Rehabilitation Tehran University of Medical Sciences Tehran Iran; ^9^ Non‐Communicable Diseases Research Center, Endocrinology and Metabolism Population Sciences Institute Tehran University of Medical Sciences Tehran Iran; ^10^ Endocrinology and Metabolism Research Center, Endocrinology and Metabolism Clinical Sciences Institute Tehran University of Medical Sciences Tehran Iran

**Keywords:** Birjand, elderly, sleep quality, social capital

## Abstract

**Purpose:**

Given the growing elderly population in developing countries like Iran, and the significance of social capital and sleep quality in the lives of the elderly, this study aimed to explore the relationship between social capital and sleep quality in the elderly community.

**Patients and Methods:**

This cross‐sectional study was conducted on elderly individuals in Birjand city, Iran, as part of a cohort study. Sampling was carried out by selecting eligible participants based on a simple random entry criterion. To gather data, we used questionnaires on social capital and Pittsburgh Sleep Quality, which were validated for reliability. The questionnaires were administered through interviews. The data was analyzed using STATA software and appropriate descriptive and analytical tests.

**Results:**

A total of 1348 senior citizens, with an average age of 69.73 (±7.53), were involved in the research conducted. The results of this study revealed a significant association (*p* < 0.05) only in the fifth quintile of social capital when compared to the lowest level. These results also show that the social capital in the group that sleeps more than 6 h is stronger than the group that sleeps less than 6 h. Also, married people in each group with less sleep (RR = 0.92) or more than 6 h (RR = 0.66) had a better condition than single people.

**Conclusion:**

Improving healthcare availability, implementing intervention strategies such as community and public health initiatives, promoting healthier sleep habits, and enhancing social support are potential methods to prevent low social involvement and, consequently, poor quality of sleep‐in older people.

## 1 Introduction

The world continues to experience a sustained change in the age structure of the population, driven by increasing life expectancy and decreasing in the number of births. People are living longer lives, and both the share and the number of older people in the total population are increasing rapidly. Globally, there were 727 million persons aged 65 years or over in the world in 2020. Over the next three decades, the global number of older persons is projected to more than double, reaching over 1.5 billion in 2050. All regions will see an increase in the size of their older population between 2020 and 2050. population aged 65 years or over is expected to increase from 9.3 per cent in 2020 to 16.0 per cent by 2050 [1]. The phenomenon of aging has always been associated with various physical and mental disorders, and researchers in the fields of medicine, psychiatry, psychology, and sociology have discussed and investigated them [2].

Despite the huge amount of money spent annually on health promotion programs, the importance of many determining factors in this field, such as social relations, mutual trust between citizens and social capital, is neglected [3]. Social capital is one of the most important and basic factors affecting health. This concept manifests as social norms and networks that allow individuals to engage in collective activities and reap reciprocal advantages through participation. Increasing social capital appears to have a significant impact on the quality of life and the health of older individuals [4, 5].

Insomnia is one of the most common complaints reported by the elderly, which is characterized by difficulty falling asleep or frequent awakenings. In a survey, it is estimated that 40%–50% of adults over 60 years of age have reported sleep disorders [6] and one of the common problems of old age is sleep disorder, and this disorder is related to many other diseases and disorders. Poor quality sleep, after headaches and digestive disorders, is the third most common problem among the elderly, which is one of the most common complaints and the reason they go to doctors and psychologists [7]. Studies comparing the sleep of elderly people with other people have shown that elderly people spend less time in slow wave sleep or deep sleep. Brain function and systemic physiological processes such as metabolism, cardiovascular and immune system function usually change according to the amount of this factor. The low quality of sleep affects recovery, memory, mood and daily performance of a person. This factor usually faces more changes in patients with psychiatric disorders. In general, sleep deprivation causes damage to the body's systems and also leads to mild cognitive impairment, increased risk of type 2 diabetes and cardiovascular disease, and disruption of the body's immune system [8]. One of the best way to assess the effect of risk factors on the prevalence of common disease in various societies, is through population cohort studies [9]. While there has been extensive study in various areas of health, the associations between quality of sleep and social capital are not as well understood [10, 11]. Considering the aforementioned issues and the role of social capital in promoting health, as well as the matter of enhancing sleep quality in the elderly, the researchers of this study opted to examine the relevance between these two factors.

## 2 Materials and Methods

### 2.1 Study Design and Sampling

In this cross‐sectional study, the basic data of Basic Linear Algebra Subprograms (BLAS) was examined. In October 2018, the BLAS began its cohort study of the elderly population in Birjand, Iran. We previously reported a detailed sampling process [12, 13]. Sampling was done based on multiple levels clusters using random sampling method. Initially, the town was divided into 70 blocks using the Geographic Information System (GIS) and taking into account the elderly population (age 60 years and older). The weight of the age categories 60–69, 70–79, and 80 years old and above was used to conduct sampling in each block. Simple random sampling was used to conduct sampling in rural areas based on a medical registration code. A group of 40 eligible participants was chosen in each of the 10 villages surrounding Birjand. inclusion criteria included: participants who were 60 years old or older and willing to take part and individuals who were bedridden or had a severe illness with a life expectancy of less than 6 months excluded of the study.

#### 2.1.1 Ethics Approval and Consent to Participate

This study is based on a research project that was approved by the Ethics Committee of Tehran & Birgand University of Medical Sciences, with the ethical code IR. TUMS. EMRI. REC. All procedures conducted in this study followed the ethical standards set by the institutional and/or national research committee, as well as the 1964 Helsinki Declaration and its subsequent amendments or equivalent. All subjects and/or their legal guardians provided written informed consent. In cases where there was significant cognitive impairment, informed consent was obtained from one of their closest relatives. For illiterate participants, informed consent was obtained from their legal guardians.

### 2.2 Data Collection Tools

The data collection tool was a questionnaire which was completed by the study participants.

#### 2.2.1 Demographic Information

Included questions about gender, age, marital status, education level, occupation, and living situation. It also gathered information from participants and their close relatives about any previously diagnosed medical conditions and how long they had been dealing with these health issues.

#### 2.2.2 Social Capital Questionnaire

A comprehensive questionnaire consisting of 69 items was utilized to evaluate social capital [14]. This questionnaire examined various levels of social capital, including immediate family, extended relatives, neighbors, colleagues, friends, individuals from similar ethnic and religious backgrounds, and the general public. The questionnaire covered various domains, including voluntary involvement, collective engagement, trust, social unity, social support, participation in community associations, and overall social interactions. The questionnaire was developed by a diverse team of experts who were involved in the Urban Heart Study conducted in Tehran. The validity and reliability of this social capital assessment tool were previously evaluated [15].

#### 2.2.3 Sleep Quality Questionnaire

To assess sleep quality, we employed the Pittsburgh Sleep Quality Index (PSQ). This survey consists of 19 self‐report questions across seven categories. The questionnaire achieved acceptable levels of internal consistency, test‐retest reliability, and validity. A global PSQI score > 5 yielded a diagnostic sensitivity of 89.6% and specificity of 86.5% (kappa = 0.75, *p* < 0.001) in distinguishing good and poor sleepers [16].

### 2.3 Data Analysis

The participants were divided into different groups based on their sleep duration for adults (around 6–8 h) [17]: less than or equal to 6 h per day and more than 6 h per day. We used one‐way analysis of variance (ANOVA) to compares the means of independent groups to determine whether there is statistical evidence that the associated variables mean is significantly different. Also, we used the Chi‐square test to examine the distribution of participants based on general demographic characteristics in each sleep duration category. To measure sleep quality, we calculated multivariable odds ratios and 95% confidence intervals. Confounder used in these models included age, gender, Body mass index (BMI) categories, smoking status, physical activity, consumption of carbonated beverages, and dairy consumption. All variables, except for age, gender, and smoking status, were treated as categorical variables. *p*‐values less than 0.05 were considered statistically significant. These statistical analyses were performed using STATA software version 14 for both males and females.

## 3 Results

### 3.1 Population Characteristics

A total of 1,348 people took part in the research, with 697 (51.71%) being female. The average age of the participants was 69.73 (7.53). Out of the total participants, 245 (18.2%) were not married, and 606 (44.96%) had no formal education (Table 1). As the results show, the sleep duration between the two groups with good and bad sleep was significant (*p* = 0000), that is, in the people who were in the bad sleep group, the majority of people (75.39%) had less than 6 h of sleep, and while in the group with good sleep, the majority of people (62.41%) in the group with good sleep had 6 to 8 h (Table 1). The majority of the participants were overweight (515, 38.20%). There were 485 (35.98%) individuals with a normal BMI, 285 (21.14%) who were obese, and 63 (4.67%) who were underweight. A total of 977 (76.60%) individuals had at least one chronic disease, and 207 participants were taking multiple medications (Table 2).

**Table 1:** Demographic variables of the elderly participating in the study.

PV	Sleep quality				Variables	
	Bad		Good			
	Percent	Frequency	Percent	Frequency		
0.57	57.94	186	55.60	571	60–69 years	Age
	31.15	100	31.25	322	70–79 years	
	10.90	35	13.05	134	80+ years	
	100	321	100	1027	Total	
0.00	63.55	204	48.00	493	Female	Sex
	36.45	117	52.00	534	Male	
	100	321	100	1027	Total	
0.0323	45.79	147	44.69	459	Illiterate	Education level
	38.01	122	36.90	379	Lower than diploma	
	10.59	34	9.64	99	Diploma	
	5.61	18	8.76	90	Academic	
	100	321	100	1027	Total	
0.002	31.15	100	39.34	400	Retired	Job status
	10.28	33	13.34	137	Employed	
	54.52	175	42.06	432	Housekeeper	
	4.05	13	5.26	54	Unemployed	
	100	321	100	1027	Total	Marital status
0.034	21.81	70	17.06	175	Not married	
	78.19	251	82.94	851	Married	
	100	321	100	1027	Total	
0.000	75.39	242	32.15	330	Less than 6 h	Sleep duration
	21.50	69	62.41	641	6–8 h	
	3.12	10	5.45	56	More than 8 h	

**Table 2:** Physical condition and disease of the elderly participating in the study.

Sleep quality				Variables	
Bad		Good			
Percent	Frequency	Percent	Frequency		
81.93	263	85.49	878	No polypharmacy	Drug number
18.07	58	14.51	149	Polypharmacy	
50.00	152	57.49	560	Less than two morbid	Multimorbidity
50.00	152	45.51	414	Multimorbidity	
100	304	100	974	Total	
68.54	220	74.20	762	0	DM final
31.46	101	25.80	265	1	
100	321	100	1027	Total	
45.48	146	42.84	440	0	Final HTN
54.52	175	57.16	587	1	
100	321	100	1027	Total	
97.81	313	99.12	1015	Has not	COPD
2.19	7	0.88	9	Has	
100	320	100	1024	Total	
58.13	186	86.91	890	No depressed mood	Depressed mood
41.88	134	13.09	134	Depressed mood	
100	320	100	1024	Total	
41.43	133	49.07	504	0	Abdominal obesity
58.57	188	50.93	523	1	
100	321	100	1027	Total	
35.51	114	36.12	371	Ideal body weight	BMI
4.36	14	4.77	49	Low body weight	
32.71	105	39.92	410	Overweight	
27.41	88	19.18	197	Obese	
100	321	100	1027	Total	
91.88	294	95.31	975	Has not	Asthma
8.13	26	4.69	48	Has	
100	320	100	1023	Total	
87.85	282	88.90	913	Has not	CHF
12.15	39	11.10	114	Has	
100	321	100	1027	Total	

*Note:* *DM: Diabetes Mellitus, **HTN: hypertension, ***COPD: Chronic Obstructive Pulmonary Disease, ****BMI: Body Mass Index, *****CHF: Congestive Heart Failure.

### 3.2 Evaluation of the Impacts of Social Capital on Sleep Quality

The multivariable stepwise binary logistic regression utilized to evaluate the influence of the overall social capital score on sleep quality (Table 3). The crude model revealed a significant association (*p* < 0.001) only in fifth quintiles of social capital when compared to the lowest level. Furthermore, when adjusting for potential confounding factors, only the highest level of social capital (fifth quintile) in model 1, model 2, model 3, and the final model demonstrated a significant association compared to the lowest level (*p* < 0.05). (Table 3) The final model was determined using the backward elimination method. These results were also observed in the final model with depression in fifth quintiles, OR: 0.87, 95% CI: 0.39 to 0.94, *p* < 0.001.

**Table 3:** Multivariable logistic regression of the effect of social capital on sleep quality.

	**Quintiles of social capital**	**Sleep quality dual**	
		OR (95% CI)	*p*‐value
Crude model	First	1	
	Second	0.82 (0.55–1.21)	0.325
	Third	0.82 (0.55–1.23)	0.352
	Fourth	0.69 (0.46–1.03)	0.074
	Fifth	0.48 (0.31–0.74)	0.001
Model 1	First	1	
	Second	0.84 (0.56–1.25)	0.410
	Third	0.87 (0.58–1.30)	0.517
	Fourth	0.75 (0.49–1.13)	0.175
	Fifth	0.56 (0.36–0.88)	0.012
Model 2	First	1	
	Second	0.85 (0.57–1.27)	0.439
	Third	0.87 (0.58–1.30)	0.515
	Fourth	0.75 (0.49–1.13)	0.175
	Fifth	0.56 (0.36–0.87)	0.011
Model 3	First	1	
	Second	0.86 (0.58–1.29)	0.487
	Third	0.88 (0.59–1.32)	0.551
	Fourth	0.76 (0.50–1.16)	0.212
	Fifth	0.56 (0.36–0.88)	0.013
Model 4	First	1	
	Second	1.05 (0.67–1.63)	0.826
	Third	1.14 (0.73–1.78)	0.540
	Fourth	0.96 (0.61–1.52)	0.888
	Fifth	0.73 (0.45–1.19)	0.218
Final model with depression	First	1	
	Second	0.98 (0.64–1.50)	0.960
	Third	1.02 (0.67–1.57)	0.901
	Fourth	0.90 (0.58–1.39)	0.638
	Fifth	0.72 (0.45–1.14)	0.169
Final model without depression	First	1	
	Second	0.87 (0.58 to 1.30)	0.519
	Third	0.91 (0.61 to 1.36)	0.661
	Fourth	0.80 (0.53 to 1.22)	0.313
	Fifth	0.60 (0.39 to 0.94)	0.027

*Note:* Model 1: controlled for demographic variables (age, gender), Model 2: controlled for extra physical characteristics (age, gender, education, marital status), Model 3: controlled for additional chronic conditions (age, gender, education, marital status, BMI, abdominal obesity), Model 4: controlled for additional lifestyle factors (age, gender, education, marital status, BMI, abdominal obesity, level of physical activity, smoking, diabetes, hypertension, COPD, asthma, congestive heart failure, multiple chronic conditions, depressive symptoms, hypnotic drugs).

The results in Table 3 also indicate that in comparison to the reference [1], the best odds ratio was observed in the fifth quintile in the initial model (0.48), suggesting that a higher social capital status of 0.52 had an impact on the likelihood of better sleep quality. The odds ratio in only model 4, quintiles 2 and 3, and in the final model, showed a worse sleep condition in quintile 3 (more than one). In the other models, a positive relationship was observed.

Table number 4, considering those who slept 6 to 8 h as the baseline group, shows that in people who slept less than 6 h, the amount of sleep quality in quantiles 3 and 4 with 95% confidence (i.e., adjusting lifestyle factors and the state of chronic diseases) relative risk is higher (1.23, 1.01) than the group that sleeps more than 6 h a day. These results show that the social capital in the group that sleeps more than 6 h is stronger than the group that sleeps less than 6 h. But in those who slept more than 6 h a night, the relative risk was not high in any of the quintiles) RR < 1).

**Table 4:** The survey of social capital in the elderly who slept more or less than 6 h' day and night.

Sleep duration	Quintiles of social capital	RRR	Std. err	*z*	*p* > |z|	[95% CI]
Lower than 6 h	First	1				
	Second	0.78	0.15	−1.20	0.23	0.53–1.16
	Third	1.23	0.24	1.08	0.28	0.83–1.83
	Fourth	1.01	0.20	0.07	0.94	0.68–1.49
	Fifth	0.97	0.19	−0.12	0.90	0.65–1.45
More than 6 h	First	1				
	Second	0.34	0.15	−2.34	0.019	0.14–0.84
	Third	0.58	0.24	−1.30	0.19	0.25–1.31
	Fourth	0.35	0.16	−2.18	0.03	0.13–0.90
	Fifth	0.39	0.19	−1.93	0.05	0.15–1.01

The results of Table 5 show the status of social capital in demographic variables, taking into 95% confidence interval, as it can be seen in the results of this table, those who slept less than 6 h on gender, women have a better status in social capital than men (RR = 0.72). Also, those who were over 80 years old and slept less than 6 h had a better condition (RR = 0.71) than people under 80 years old (RR = 1.20), but in those who slept more than 6 h in people under 80 years old, they showed a better condition (RR = 2.46). Also, married people in each group with less sleep (RR = 0.92) or more than 6 h (RR = 0.66) had a better condition than single people.

**Table 5:** Associations of sleep pattern with some demographic factors.

[95% CI]	*p* > |z|	*z*	Std. err	RRR	Variables		Sleep duration
0.51–1.01	0.061	−1.87	0.12	0.72		Sex	Lower than 6 h
0.90–1.58	0.19	1.28	0.17	1.20	70–79	Age	
0.45–1.11	0.13	−1.49	0.16	0.71	80+		
1.02–1.82	0.034	2.12	0.20	1.36	Lower than diploma	Education	
1.11–2.74	0.016	2.41	0.40	1.74	Diploma		
0.88–2.27	0.14	1.44	0.34	1.41	Academic		
0.58–1.51	0.80	−0.24	0.22	0.94		Marital	
0.73–2.12	0.41	0.81	0.33	1.24		Living arrangement	
0.65–2.48	0.47	0.72	0.43	1.27	Low body weight	BMI	
0.89–1.67	0.20	1.26	0.19	1.22	Over weight		
1.07–2.44	0.20	2.33	0.33	1.62	Obese		
0.58–1.21	0.36	−0.91	0.15	0.84		Abdominal obesity	
0.86–1.63	0.28	1.06	0.19	1.18		DM final	
0.68–1.23	0.57	−0.56	0.13	0.91		Final HTN	
0.63–10.56	0.18	1.33	1.85	2.59		COPD	
0.76–2.28	0.32	0.99	0.37	1.32		Asthma	
0.89–2.03	0.15	1.43	0.28	1.35		CHF	
0.72–1.46	0.86	0.17	0.18	1.03		Multi morbidity	
1.31–2.48	0.00	3.65	0.29	1.80		Depressed mood	
1.43	0.56	−0.57	0.22	0.86		Cons	
0.44–1.62	0.61	−0.50	0.28	0.84		Sex	More than 6 h
0.79–3.05	0.20	1.28	0.53	1.55	70–79	Age	
1.16–5.21	0.01	2.35	0.94	2.46	80+		
0.35–1.41	0.32	−0.98	0.24	0.70	Lower than diploma	Education	
0.57–4.10	0.39	0.86	0.77	1.53	Diploma		
0.35–2.17	0.22	−1.22	0.29	0.27	Academic		
0.25–1.72	0.39	−0.85	0.32	0.66		Marital	
0.41–3.35	0.75	0.31	0.62	1.18		Living arrangement	
2.06–11.36	0.00	3.63	2.10	4.84	Low body weight	BMI	
0.30–1.39	0.27	−1.10	0.25	0.65	Over weight		
0.54–2.64	0.64	0.46	0.48	1.20	Obese		
0.34–2.03	0.68	−0.40	0.38	0.83		Abdominal obesity	
0.51–2.52	0.73	0.33	0.46	1.14		DM final	
0.44–2.00	0.88	−0.15	0.36	0.94		Final HTN	
067–0.37	0.11	1.57	5.10	4.99		COPD	
0.33–2.78	0.94	−0.06	0.63	0.96		Asthma	
0.33–2.78	0.94	−0.06	0.52	0.96		CHF	
0.48–2.76	0.75	0.32	0.51	1.15		Multi morbidity	
0.73–2.75	0.29	1.04	0.47	1.42		Depressed mood	
0.04–0.47	0.00	−3.21	0.88	0.14		Cons	

Abbreviation: RRR: Relative Risk Regression.

## 4 Discussion

Numerous studies have established a strong correlation between social factors, such as social capital, and the overall well‐being of individuals [18, 19]. Social capital may play a crucial role in sleep disorders due to its impact on emotional perception and psychological state [20].

According to our findings, after accounting for potential factors that could influence the results, we observed a significant relationship between the highest social capital level (fifth quintile) and quality of sleep. This could be attributed to the support and sense of belonging provided by social connections, which can reduce stress and enhance safety feelings. insufficient social capital has been associated with inadequate quality of sleep, heightened sleep disturbances, and an elevated susceptibility to disorders of sleep [11]. Individuals experiencing feelings of isolation may encounter difficulties in both initiating and maintaining sleep due to heightened levels of stress and anxiety. Moreover, those with strong social ties tend to experience better sleep due to a heightened sense of safety and security. In summary, these findings underscore the significance of social supports and connection in fostering a healthy sleep pattern [11, 21].

There is a dearth of research that investigates the potential connection between quality of sleep social capital among older individuals [22, 23]. Nevertheless, it is crucial to acknowledge that sleep issues are more common among older adults in comparison to younger individuals. Additionally, research has linked poor quality of sleep among older individuals with impaired cognitive functioning [24]. Robbins et al. conducted a study to investigate the correlation between various aspects of sleep, including daytime sleepiness, sleep duration, and insomnia and social capital. Their findings showed that although not all connections between components of social capital and sleep were statistically significant, there was an overall tendency suggesting a link between insufficient social capital and inadequate sleep quality [21]. In a separate cohort study among old people in Britain, it was discovered that increased exposure to positive support was linked to improved quality of sleep. This suggests that having positive social relationships can contribute to better sleep quality [25]. According to Masoudnia's research findings, individuals who possessed stronger and more extensive social connections, along with strong relations with religious organizations, friends, and family, experienced higher sleep quality levels [26]. Additionally, a study by Win et al demonstrated that perceived trust in social capital was linked to individuals' health. they proposed that fostering social capital could potentially enhance sleep quality among Japanese women [27]. Furthermore, Young et al. put forth the argument that American adults who had lower levels of network social capital experienced poorer sleep quality [28]. These studies underscoring the significance of positive social connections in fostering improved sleep outcomes.

One possible mechanism through which social capital and social relationships can affect sleep quality is through susceptibility feeling. For instance, Hawkley and Cacioppo's research indicated that the experience of feeling susceptible can lead to heightened vigilance, ultimately leading to a decline in sleep quality [29]. Social capital can help mitigate environmental and psychosocial stressors that disrupt sleep by fostering mental well‐being through social support, reciprocal trust, extensive social networks, reciprocal actions [30]. Furthermore, residing in a community characterized by trustworthiness can cultivate feelings of safety and security, thereby facilitating easier sleep onset. Sleep is acknowledged as a behavior influenced by psychological factors, and changes in these factors resulting from stress can adversely affect sleep quality [31]. For example, according to Xiao et al., their research revealed that social capital had a significant impact on anxiety and stress levels, with anxiety affecting directly quality of sleep [20]. Furthermore, individuals with more social capital often have access to spiritual or material support from others, which can help reduce stress levels. Stress can negatively impact sleep quality as it can lead to mental pressure, physical tension, excessive focus on sleep, and increased sensitivity to the environment of sleeping [32]. Moreover, investigations offer valuable insights into the connection between quality of sleep and social cohesion [33].

Our results show that the social capital in the group that sleeps more than 6 h is stronger than the group that sleeps less than 6 h. Old people who have limited social involvements and few social ties are likely to engage in less physical activity, which may have an impact on their sleep quality, too [34]. Moreover, individuals who have limited participation in activities in social and possess low levels of social trust as part of their social capital may encounter feelings of boredom and a stimulation deficiency in day, which can make it more challenging to sleep during the night [35]. Additionally, older adults who have limited social engagement may encounter feelings of depression and loneliness, that may disorder sleep patterns [36]. Furthermore, insufficient engagement and connectivity within social circles have been linked to a higher occurrence of chronic health ailments among elderly individuals, and these health issues can subsequently worsen the overall quality of sleep [37]. These can result in adverse effects and potentially develop into dependency as time goes on [38]. Takahashi and colleagues discovered that individuals with stronger social connections in their social environment had better sleep quantity [39]. Alhasan et al's research revealed that individuals with low social attachments tended to have shorter duration of sleep [40]. Nutakor et al's study found that poor social capital was linked to higher short sleep risk [41]. Several investigations suggest that sleep is influenced by psychological factors and the stress‐relieving impact of a supportive and secure social environment on asleep status [32, 42]. When individuals experience stress, they often feel mental and physical tension [43], becoming more vulnerable to their sleeping environment, potentially impacting duration of sleep. 32 For older adults, social integrations are essential for their health condition, which in turn can enhance better sleep.

When examining the connection between demographic characteristics, social capital, and sleep duration in the elderly, there was a limited availability of articles on this topic. This study was constrained by the lack of literature, which restricted further discussion on the subject. As a result, comparisons in this area were based on only a small number of existing studies.

Our findings indicated that women with less than 6 h of sleep had higher levels of social capital compared to men. It was suggested that women may benefit from social connections when participating in health‐related behaviors, as well as utilizing social support more effectively than men, which could impact their sleep. Conversely, men may involve in different social activities that they do not necessarily need social cohesion. Previous research has also found stronger associations between aspects of the sleep dimensions and social environment in women compared to men [44, 45]. Studies by Young et al and Win et al also highlighted gender differences in the relationship between social capital and sleep duration [27, 28]. Moreover, women may have developed better strategies for dealing with sleep deprivation, enabling them to remain engaged in social activities and uphold their social connections despite their lack of sleep. Additionally, investigation by Kim and Woo demonstrated that perceived trust in social capital was related to self‐rated health through duration of sleep among women, suggesting that promoting social capital could improve sleep duration for this demographic [46]. According to socioecological theories, previous research suggested that women's increased susceptibility to the social environment is attributed to the specific stressors they encounter in their daily social roles and how they are affected by support network. However, despite these considerations, previous studies focusing on sleep status and social capital did not display any significant differences based on gender [47, 48]. While various mechanisms have been proposed to explain the link between duration of sleep and social capital, it was until now unclear whether there were sex differences in these relations. According to these conflicting results, more research is necessary to indicate if these findings regarding sex differences are consistent to identify potential underlying factors.

Our results showed that those who were over 80 years old and slept less than 6 h had a better social capital condition than people under 80 years old. However, the assessment of age and sleep status is confounded and influenced by various factors. It is important to consider age as a factor when studying the effects of sleep on social capital. Further research is needed to better understand the complex relationship between age, sleep duration, and social capital. In the elderly population, interpreting data is specially challenging due to the presence of comorbid situations. Polysomnographic findings suggest that sleep time decrease with age [49]. Elderly population may be more affected by their social environment compared to younger population, possibly due to a decreased network relationship resulting from factors such as loss of loved ones, retirement, and health compromised [50]. Overall, elderly population tend to shorter sleep than young population, indicating negative relations between duration of sleep and age. Sleep situation changes can be influenced by lifestyle and medical disorders [51]. It's important to note that more investigations are required to understand the basic procedures driving such as findings. While there aren't more investigations available for comparison, these results suggest the potential benefit of intervening in sleep patterns earlier to health improvement.

The study revealed that married individuals, regardless of their sleep duration, exhibited better social capital condition compared to single individuals. It indicates that being married may be associated with better social connections and support networks. There is limited information available on the distribution of social capital based on demographic factors such as marital status, with only a few studies presenting relevant results. Married couples frequently share their lives with a partner, leading to shared social connections and networks. This can expand their social circle, making it more varied and extensive. Additionally, the stability that comes with marriage can foster a heightened sense of security and belonging, positively influencing an individual's capacity to establish and sustain social connections. Based on the Stone and Hughes study, individuals who have partner tend to have higher social capital compared to people living alone and with no partner [52]. Similarly, Nieminen et al indicated that married individuals appeared to have higher levels of social capital [53]. Based on this evidence, it can be inferred that social capital may serve as an indicator of overall health. These findings could be valuable for related organizations in focusing their efforts on individuals with social exclusion high risk.

Improving healthcare availability, applying interventional planning like community and public plans, promoting habit of healthier sleep, and promoting social supports are possible methods to prevent the low social involvements and consequently poor quality of sleep‐in old people. Experimental studies can be designed to explore whether improving the levels of social capital among older people leads to benefits in sleep status. More research is needed to fully understand the mediating or moderating effects of social capital on sleep quality. Therefore, it can be inferred that decreasing social isolation and improving social capital among elderly population could have significant impacts on their situation of their sleep. Design and use health promotion interventions like increasing accessibility of healthcare, community educational interventions, and improving social support are suitable methods to evaluate the relationships of social factors situation and their relations to short sleep duration among elderly population. These efforts could potentially lead to improved overall well‐being and health outcomes for older adults.

## 5 Conclusion

The results revealed that the social capital of the group that slept more than 6 h is stronger than that of the group that slept less than 6 h. Design and use health promotion interventions like increasing accessibility of healthcare, community educational interventions, and improving social support are suitable methods to evaluate the relationships of social factors and their relationships to short sleep duration among the elderly population. These actions could improve the overall well‐being and health outcomes of older adults.

## Author Contributions

**Maryam Mohammadi:** writing – original draft, writing – review and editing. **Sahar Mohammadnabizadeh:** writing – review and editing. **Mehdi Varmaghani:** writing – review and editing, software. **Mitra Moodi:** conceptualization, project administration. **Farshad Shrifi:** conceptualization, investigation, methodology, formal analysis. **Matin Shirazinia:** formal analysis, funding acquisition. **Hossein Fakhrzadeh:** conceptualization, visualization. **Moloud Payab:** conceptualization, supervision, data curation. **Masoumeh Khorashadizadeh:** conceptualization, investigation, supervision. **Shohreh Naderimagham:** conceptualization, project administration, writing – original draft, supervision, validation.

## Conflicts of Interest

The authors declare no conflicts of interest.

## Transparency Statement

The lead author Shohreh Naderimagham affirms that this manuscript is an honest, accurate, and transparent account of the study being reported; that no important aspects of the study have been omitted; and that any discrepancies from the study as planned (and, if relevant, registered) have been explained.

## Data Availability

The data that support the findings of this study are available from the corresponding author upon reasonable request. Data will be provided by the corresponding author upon request.
